# CoMFA, CoMSIA and Eigenvalue Analysis on Dibenzodioxepinone and Dibenzodioxocinone Derivatives as Cholesteryl Ester Transfer Protein Inhibitors

**DOI:** 10.3390/molecules13081822

**Published:** 2008-08-22

**Authors:** Xu-qiong Xiong, Dong-mei Zhao, Peng-fei Bu, Yang Liu, Jin-hong Ren, Jian Wang, Mao-sheng Cheng

**Affiliations:** Key Lab of New Drugs Design and Discovery of Liaoning Province, School of Pharmaceutical Engineering, Shenyang Pharmaceutical University, Shenyang, 110016, China; E-mails: marywind@gmail.com (Xu-qiong Xiong); Dongmeiz-67@163.com (Dong-mei Zhao); bpfiloveyou@sina.com (Peng-fei Bu); Ly_99@sina.com (Yang Liu); sunny_rjh@163.com (Jin-hong Ren); wjmed@126.com (Jian Wang)

**Keywords:** CoMFA, CoMSIA, eigenvalue analysis, dibenzodioxepinone and dibenzodioxocinone analogues, cholesteryl ester transfer protein inhibitors

## Abstract

CoMFA, CoMSIA and eigenvalue analysis (EVA) were performed to study the structural features of 61 diverse dibenzodioxepinone and dibenzodioxocinone analogues to probe cholesteryl ester transfer protein (CETP) inhibitory activity. Three methods yielded statistically significant models upon assessment of cross-validation, bootstrapping, and progressive scrambling. This was further validated by an external set of 13 derivatives. Our results demonstrate that three models have a good interpolation as well as extrapolation. The hydrophobic features were confirmed to contribute significantly to inhibitor potencies, while a pre-oriented hydrogen bond provided by the hydroxyl group at the 3-position indicated a good correlation with previous SAR, and a hydrogen bond acceptor may play a crucial role in CETP inhibition. These derived models may help us to gain a deeper understanding of the binding interaction of these lactone-based compounds and aid in the design of new potent compounds against CETP.

## Introduction

Cholesteryl ester transfer protein (CETP) is an important glycoprotein in human plasma that mediates the transfer of neutral lipids among lipoproteins. To date, the role of CETP in coronary heart disease (CHD) is still not fully understood. Some consider it detrimental toward CHD by lowering atheroprotective high density lipoprotein-cholesterol (HDL-C) and raising proatherogenic very low density lipoprotein-cholesterol (VLDL-C) and low density lipoprotein-cholesterol (LDL-C). However, other researchers contend that CETP has beneficial effects by facilitating cholesterol removal through the reverse cholesterol transport pathway. Although there continues to be debate on the issues surrounding CETP [1,2,3,4,5], various reversible and irreversible CETP inhibitors have already been reported [6,7,8,9,10,11,12]. Furthermore, Roche’s JTT-705 [10] and Merck’s anacetrapib [12] are both entering phase III clinical trials to treat Coronary Heart Disease (CHD) or CHD Risk-Equivalent Disease. All this shows that the development of safe and effective CETP inhibitors are potential for a novel and important class of drugs that combat lipidaemia.

**Figure 1:**
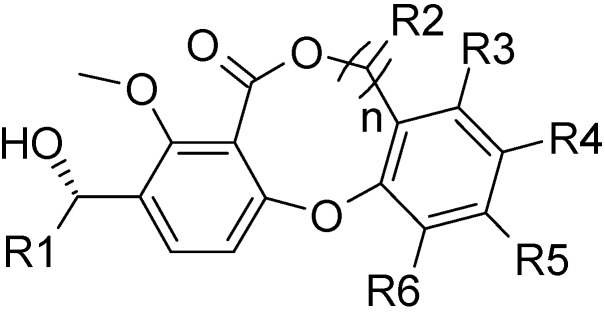
The structural core of lactone-based derivatives.

Bayer reported a series of lactone-based compounds (Figure 1) as attractive CETP inhibitors [7], but no data about their mechanism(s) of action have been reported. The crystal structure of CETP [13] reveals the most unusual structural feature of the binding site of neutral lipids - a long tunnel (60 Å) with a very large volume (2,560 Å^3^). Further, the tunnel is lined with hydrophobic amino acid side chains. All of these features indicate a large degree of uncertainty with the results of the docking studies. Consequently, three-dimensional quantitative structure-activity relationship (3D-QSAR), a ligand-based approach, may be a more informative way to investigate a variety of receptor-ligand interactions in this challenging system. 

n=0 or 1

Herein, we present a 3D-QSAR study to investigate the correlation of dibenzodioxepinone and dibenzodioxocinone derivatives with the inhibition of CETP by employing comparative molecular field analysis (CoMFA), comparative molecular similarity indices analysis (CoMSIA), and eigenvalue analysis (EVA). To the best of our knowledge, the present studies represent the first comprehensive 3D-QSAR investigation of the CETP inhibition by lactone-based compounds and the resulting models should help to understand the binding interaction of these agents and offer utility in the rational design of more effective and specific CETP inhibitors. This may aid in determining whether they could represent a novel therapeutic approach toward treating CHD patients with low HDL-C levels.

## Results and Discussion

A data set of 61 diverse lactone-based analogues was selected as a training set to derive the conventional CoMFA, CoMSIA and EVA models; an additional 13 compounds were used to test the accuracy of these models. 

### CoMFA results

We first investigated the effect of varying parameters on the derived models using leave-one-out cross-validated PLS analysis. The best model yielded a q^2^ value of 0.666, and the optimal combination of parameters was identified as (1) a column filtering energy cutoff at 4.18 kcal/mol, (2) a sp^3^ carbon atom with +1 charge as probe, and (3) a 2.0 Å grid spacing. Since q^2^ may vary as much as 0.5 units toward a different orientation or placement of the aligned molecules, an all-orientation search (AOS) and an all-placement search (APS) were applied to identify that with the highest q^2^ value. The optimal orientation was derived from AOS, with a q^2^ value of 0.699. With the steric/electrostatic field cutoff at 20.9 kcal/mol, an even better model was generated to afford a q^2^ of 0.724. Region focusing was considered as an additional strategy to improve q^2^. Nevertheless, when tested with progressive scrambling, the derived model did not provide improved results. Thus the final noncross-validated PLS analysis yielded a conventional r^2^ of 0.922, a low standard estimated error of 0.254, and a large F-value of 225.181.

### CoMSIA results

To describe the overall ligand environment in the binding pocket, all CoMSIA models were developed based on all five fields. First, a better model was obtained by the optimal settings, with a 0.2 attenuation factor, a column filtering energy cutoff of 6.27 kcal/mol, and a 2.5 Å grid spacing. This model yielded a q^2^ of 0.708. In addition, region focusing and progressive scrambling were explored as above. The difference from the CoMFA results lies in the region-focused model manifested as an improved CoMSIA model with a q^2^ of 0.740. As such, the final noncross-validated PLS analysis model was established from region-focused descriptors.

### EVA results

Unlike CoMFA and CoMSIA, EVA employs a novel alignment-free descriptor of molecular structure, not field descriptors. Based on the default parameters, the derived EVA descriptor consisted of 761 variables per structure. For a standard QSAR dataset the number of variables is so larger that PLS was used to provide a robust regression analysis. It was found that the derived EVA model was comparable, in statistical terms, to two former models (Table 1). With an optimal number of components 2, the EVA model had a higher q^2^ of 0.754, a lower conventional r^2^ of 0.834 and a higher standard estimated error of 0.367. 

**Table 1:** Summary of 3D-QSAR analyses.

	CoMFA	CoMSIA	EVA
Components	3	3	2
q^2^	0.724	0.740	0.754
Conventional r^2^	0.922	0.842	0.834
Standard error of estimate	0.254	0.361	0.367
F values	225.181	101.238	146.138
Bootstrapping			
r^2^_bootstrapping_	0.922±0.019	0.845±0.031	0.857±0.028
Standard error of estimate _bootstrapping_	0.240±0.113	0.338±0.143	0.322±0.137
Progress scrambling			
Q^2^	0.493	0.597	0.639
cSDEP	0.646	0.576	0.540
d*q*^2′^/d*r*^2^*_yy_*_′_	0.924	1.042	0.952
Predictive r^2^	0.823	0.782	0.571
Fraction			
Steric	0.594	0.204	
Electrostatic	0.406	0.166	
Hydrophobic		0.303	
Hydrogen bond donor		0.139	
Hydrogen bond acceptor		0.187	

**Figure 2:**
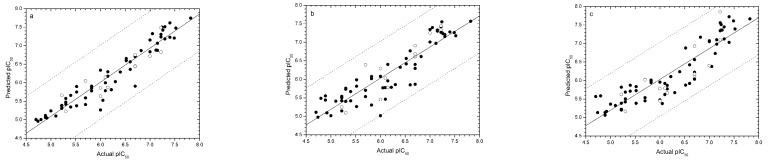
Plots of experimental and predicted pIC_50_ values of the training (●) and test (○) sets in the CoMFA (a), CoMSIA (b) and EVA (c) models. The solid line is the regression line for the training set predictions, while the dotted lines indicate the ± 1.0 log point error margins.

The predicted pIC_50_ value for each compound in the training set and its residual value are shown in Table S1 (see Supplementary data). Generally, extrapolations of up to one order of magnitude (± 1 log unit) are acceptable. Fortunately, there is no outlier in all three models (Figure 2). The largest residual in two models is that of compound **4** (0.99) in the CoMSIA model, which still falls within one log unit.

### Validation of models

Leave-one-out cross-validated PLS was introduced to generate an initial measure of the accuracy of model interpolation. The study suggested that all the derived models had a good cross-validated correlation (q^2^ > 0.6). Although the q^2^ value of the CoMFA model was lower than those of the other two models, conventional r^2^ and F-value were higher, while the standard estimated error was lower. As a result, the cross-validated PLS analysis indicated that the CoMFA model was superior to the CoMSIA and EVA models.

We further performed bootstrapping analyses to evaluate the robustness and statistical confidence of the final models. The results indicated a high confidence limit to all three models, with the CoMFA model performing better, in agreement with the above results.

Concurrently, progressive scrambling was calculated to assess the dependence of the derived model on chance correlations. All three models were well behaved, with the components for the noncross-validation analysis found optimal for each, as well as a minimal cSDEP value and a maximal Q^2^ value (Table 1). This suggests that all models are stable, with the EVA model performing best.

In order for models to be useful toward lead optimization, they must have reasonable extrapolative validity in addition to interpolative accuracy. Subsequently, an external validation was preformed on a test set of 13 compounds outside the training set to evaluate their predictive ability. All the compounds were predicted well by three models, with residuals within 1 log unit (Table 2). Moreover, the CoMFA and CoMSIA models gave good predictive r^2^ values of 0.823 and 0.782, respectively; while the predictive r^2^ value of the EVA model is lower, at only 0.571. The CoMFA model again appears to have better external predictive ability than the other two models by comparison of the residual distribution and the predictive r^2^ values.

**Table 2:** Experimental and predicted values for the test set.

Compd	Obsd pIC_50_	CoMFA		CoMSIA		EVA	
		predicted	residual	predicted	residual	predicted	residual
E3	6.00	5.64	0.36	5.45	0.55	5.45	0.55
E8	5.30	5.23	0.07	5.09	0.21	5.17	0.13
E12	5.22	5.66	-0.44	5.25	-0.03	5.62	-0.40
E22	6.15	5.87	0.28	5.78	0.37	5.67	0.48
E23	6.70	6.44	0.26	6.61	0.09	6.12	0.58
E27	5.70	6.05	-0.35	6.39	-0.69	6.02	-0.32
E32	6.00	5.86	0.14	6.29	-0.29	5.78	0.22
E39	7.22	6.82	0.40	7.34	-0.12	7.10	0.12
E40	7.00	6.72	0.28	7.25	-0.25	6.40	0.60
E49	7.22	7.49	-0.27	7.44	-0.22	7.86	-0.64
E70	6.10	6.15	-0.05	6.03	0.07	5.79	0.31
E73	6.70	6.44	0.26	6.69	0.01	6.18	0.52
E74	6.70	6.75	-0.05	6.91	-0.21	6.93	-0.23

Overall, three models are statistically significant with a high confidence level, and have a good interpolation as well as extrapolation. Further, we confirmed that the internal and external predictive ability of the CoMFA model is superior; however, the CoMSIA model shows more stable than the CoMFA model, and the EVA model shows most stable with the least external predictivity.

### 3D-QSAR contour analysis

**Figure 3:**
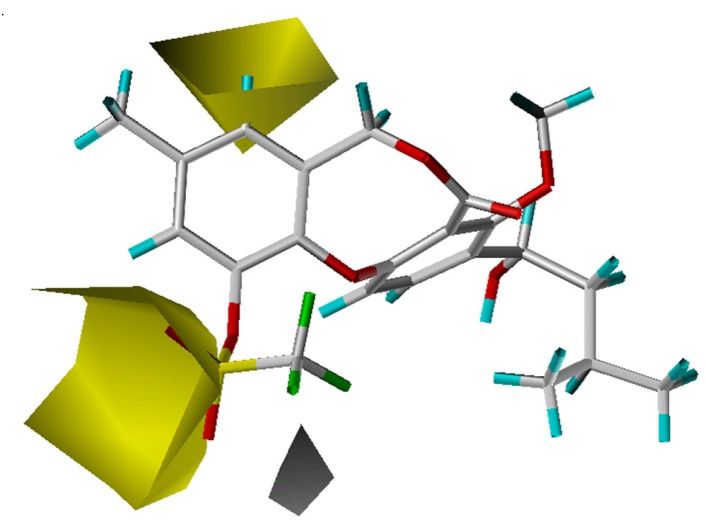
Hydrophobic maps from the CoMSIA model. Compound **63** is shown inside the field. The two yellow areas (contour level 0.02) suggest that hydrophobic substituents will result in an increase in activity, while the white region (contour level -0.01) suggests that a corresponding decrease will result.

**Figure 4:**
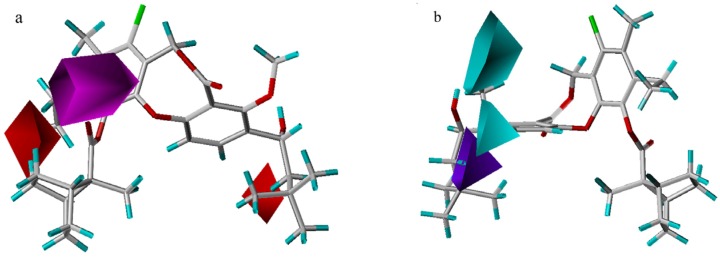
Hydrogen bond acceptor (a) and donor (b) contour maps from the CoMSIA model. Compound **55** is shown inside two fields. The magenta map (contour level 0.03) and two red maps (contour level – 0.015) denote favorable and unfavorable regions for hydrogen bond acceptor groups, respectively. Isopleths in cyan (contour level 0.005), and purple (contour level –0.03) represent favorable and unfavorable areas for hydrogen bond donor groups, respectively.

**Figure 5:**
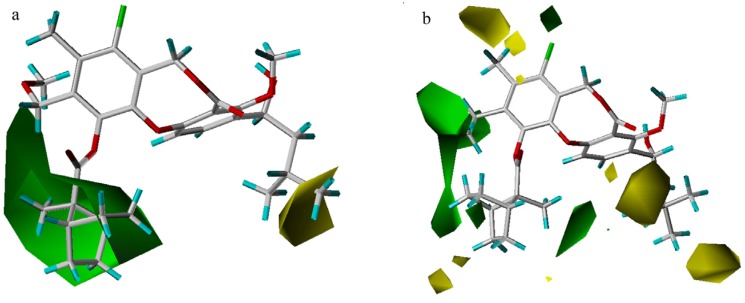
Steric maps from the CoMSIA (a) and CoMFA (b) models. Compound **51** is shown inside the CoMSIA field, while compound **55** is shown inside the CoMFA field. Green contours (contour level: CoMFA: 0.03; CoMSIA: 0.02) encompass regions that favor bulky groups, while yellow contours (contour level: CoMFA: –0.01; CoMSIA: –0.001) highlight areas that disfavor bulky groups.

**Figure 6:**
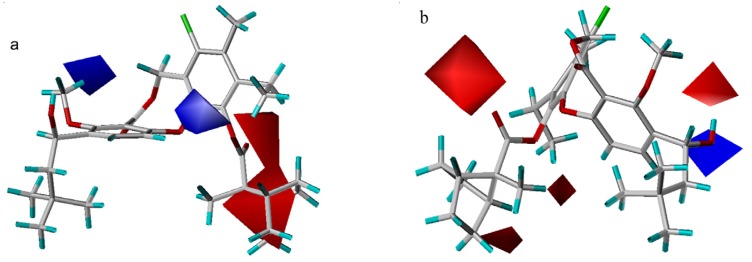
Electrostatic maps from the CoMSIA model (a) and CoMFA model (b). Compound **55** is shown inside two field models. Red isopleths (contour level: CoMFA: –0.02; CoMSIA: –0.015) define regions where electronegative groups will increase the activity, while blue contours (contour level: CoMFA: 0.03; CoMSIA: 0.012) define regions where an increase in positive potential will enhance the affinity.

The results obtained from CoMFA and CoMSIA were graphically interpreted through the stdev*coefficient contour maps (Figure 3, Figure 4, Figure 5 and Figure 6). To select the appropriate contour levels for each feature, respective histograms of the actual field values were analyzed, and contour levels that produced chemically meaningful contour maps were selected. These contour maps provide a detailed understanding of the binding mode of dibenzodioxepinone and dibenzodioxocinone derivatives, highlighting the key structural features required for the CETP affinity.

The crystal structure of CETP [13] reveals a very large hydrophobic pocket, with 44% of the amino acid residues being hydrophobic. Analysis of the fractions of five fields with the CoMSIA model confirmed that the hydrophobic effect dominantly determines the binding affinity (Table 1). As shown in Figure 3, a large hydrophobic region (yellow) and a small hydrophilic area (white) appear near the 11-position, and the most potent compounds each have a hydrophobic 11-substituent. For compound **63**, only a small portion resides in the hydrophobic region, which is actually a hydrophilic sulfonyl moiety, leading to a weak activity (pIC_50_ = 5.00) with this compound. Further, the existence of some hydrophilic group near the white hydrophilic contour has a positive effect on the activity as indicated by compounds **11** and **41** (an oxygen or nitrogen atom), which have higher CETP inhibitory activity than compounds **14** and **32**, respectively.

Hydrophobic groups at the 10-position will favor compound activity. Compound **57**, which bears a 10-ethyl group is more potent than compound **45** with a 10-chloride substituent. However, the 10-cyclopropyl group in compound **48** (pIC_50_ = 7.24) lessens the overall activity, compared to the 10-methyl substituent in compound **54** (pIC_50_ = 7.52). This may be due to the torsional force of the cyclopropyl group, which is strong enough to bias the lowest energy conformation away from the optimal bioactive conformation or cause the compound in a high-energy state. A hydrophobic group near the 8-position is also better for activity. For example, though an 11-isopentyloxy substituent in compound **3** is more hydrophobic than an 11-methoxy moiety in compound **4**, these two compounds are actually equal in activity owing to a hydrophobic ethylene group occupying the 8-position of compound **4**.

In the hydrogen bond acceptor field, a large magenta polyhedron indicates that acceptor groups are favored at the carbonyl oxygen atom in the 11-substituent or either of the other two oxygen atoms in the core ring (Figure 4a). In the hydrogen bond donor field, the two cyan areas indicate that donors are favored at the hydroxyl group at the 3-position, while one purple area denotes a region where a donor group disfavors this binding (Figure 4b). Brückner *et al*. [7] have reported that only the (*S*)-enantiomer is active (all the compounds used in this study are (*S*)-enantiomers), which may correlate with the orientation of hydrogen bond. Compound **38** is indicative of this phenomenon, in which the hydroxyl hydrogen is far from two cyan areas, unlike compound **56**. As a result, the activity of compound **38** is poor, even though the substituents in both molecules are quite similar.

In the steric contour maps (Figure 5), two models indicate a low tolerance for bulky substituents at the 3-position and a high tolerance of bulky groups at the 10- or 11-position. Compound **51** is less potent than compound **53** because an isobutyl group at R1 is partially inserted into the small yellow contour close to the 3-position, while a larger neopentyl moiety in compound **53** extends beyond the yellow contour. As exemplified by the most potent compound **55**, a large rigid ranched-bicycle at the 11-position, and an ethyl group at the 10-position both fit into the green area. However, there is a steric limit for the two small yellow contours outside the green contour near the 11-position in the CoMFA model. Compound **64** shows worse activity than compound **22**, because the n-pentylsulfonic group at the 11-position overlaps with the larger yellow contour. The two models also differ in that there exist a small green contour adjacent to the 8-position and two yellow contours near the 9-position in the CoMFA model. Though an 11-methoxy group is smaller than the isopentyloxy group, compound **4** with a larger 8-vinyl group has equal activity to compound **3**. Compound **43** exhibits lower activity than compound **3**, probably due to the presence of a larger difluoromethyl group at the 9-position, rather than a smaller methyl group.

The electrostatic contour maps are plotted in Figure 6. Both models indicate that more negatively charged 11-substituents will have a positive effect on the potency. For example, compounds **24**–**31** all exhibit lower activity due to the lack of electron density in this region. Additionally, the CoMSIA contour map shows that a positive 11-carbon or a positive 4-substituent will increase ligand binding affinity. Nevertheless, it is difficult to explain why there are two opposite contour areas close to the hydroxyl moiety at the 3-position in the CoMFA electrostatic contour plot. They may be a computational artifact.

From the above comparisons, we conclude that the priority of the 3-, 10- and 11-substituents in the CoMFA model is in good agreement with that of the CoMSIA model, further validating the reliability of the derived models.

### Graphical interpretation of EVA Results

Unlike CoMFA and CoMSIA, EVA uses 2D plots to visualize the EVA descriptor in the form of a ‘spectrum’, although this descriptor is not intended to simulate the infrared spectrum of a molecule. This permits the interpretation of the EVA descriptor by examination of the distribution of vibrations in a molecule or in a set of molecules.

**Figure 7:**
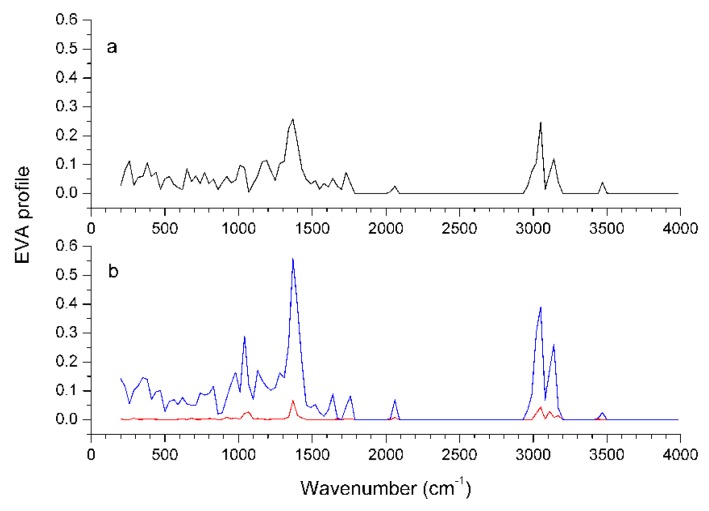
Characteristics of the EVA profiles. EVA pseudo-spectra of the weakest compound **19** (a) and the most potent compound **55** (b) are given by black and blue lines, respectively; the Discriminant Power profile of the training set is given by the red line.

The Discriminant Power profile of the EVA model is shown in Figure 7. The largest variations over the training set are contributed at frequencies centered around 1390 and 3070 cm^-1^, corresponding to C-H bending and stretching vibrations. There are prominent peaks in these two regions for the most active compound **55**, which possesses many CH_3_ or CH_2_ groups at the 3-, 10-, and 11-positions. But these peaks are sharply attenuated in the EVA profile for the least active compound **19**, which possesses much less alkyl groups at the corresponding positions. This means that a large and hydrophobic group at the 10-, or 11-position will increase CETP inhibitory activity, in accordance with the CoMFA and CoMSIA results. Nevertheless, whether in the fingerprint region (200-1500 cm^-1^) or in the functional group region (1500-4000 cm^-1^), most of the group frequencies overlap and are nonspecific, so it is difficult to correlate them with the activity.

## Conclusions

In this study, we have investigated the CoMFA, CoMSIA and EVA models based on a training set of 61 structurally diverse dibenzodioxepinone and dibenzodioxocinone derivatives, followed by validation of the results by an external test set of 13 analogues. Three models demonstrated excellent internal and external predictive ability, which was shown by several strategies including cross-validation, bootstrapping, progressive scrambling, and predictive r^2^.

Overall, the EVA model is most stable, which is not sensitive to molecular alignment and only slightly sensitive to molecular conformation. In our case, it is somewhat difficult to interpret most molecular features with the activity. And CoMFA performed either similarly or better than CoMSIA in the steric and electrostatic fields, while the CoMSIA model was more valuable for the three fields that contributed significantly to the binding with CETP (hydrophobic, hydrogen bond acceptor, and hydrogen bond donor). The CoMSIA analysis indicated that variations in the binding affinity are dominated by hydrophobic interactions, consistent with the fact that approximately 44% of the amino acid residues in CETP are hydrophobic. The orientation of the hydroxyl group in the 3-substituent plays a crucial role in determining the biological activity: the inactivity of the (*R*)-enantiomer may be a result of a lacking hydrogen bond with the hydrogen bond acceptor near the 3-position. Additionally, the CoMSIA model suggests that a hydrogen bond acceptor may have a positive effect on the potency.

The excellent correlation with several experimental studies suggests that these 3D-QSAR models are reliable, helping us to understand the binding interaction of these lactone-based compounds and providing a helpful guideline for further lead optimization. The features derived from the above models bear a close correlation with the structural variations inherent in the training set, so other structurally distinct data may likely result in diverse features causing different conclusions. In summary, although there exist conflicting viewpoints over the link between CHD and CETP inhibition, our preliminary findings may aid in identifying potent and specific CETP inhibitors that may be used to more clearly elucidate the role of CETP in atherosclerosis, and offer more significant insights into the overall pharmacology of this system.

## Experimental

All calculations were performed using SYBYL 6.91 on a Silicon Graphics Fuel workstation with IRIX 6.5 operating system.

### Data sets for analysis

All the lactone-based derivatives used for all 3D-QSAR analyses, with pIC_50_ values (-logIC_50_) varying from 4.7 to 7.82, were pooled from the work of Brückner *et al*. [7,14] Among them, 61 compounds with diverse substituents at the 3-, 7-, 8-, 9-, 10-, and 11-positions constituted the training set, and we used the same criteria to select additional 13 compounds as a test set for model validation; there was a similar distribution of activities across both sets (Table 3). An attractive feature of the selected inhibitors is the rigid three-ring dibenzodioxepinone or dibenzodioxocinone core structure, which makes them more amenable to 3D-QSAR analyses than flexible molecules.

**Table 3:** Structures and activities of all the dibenzodioxepinone and dibenzodioxocinone derivatives used for 3D-QSAR studies.

Compd	R1	n	R2	R3	R4	R4	R6	pIC_50_
1	CH_2_CH(CH_3_)_2_	0	–	H	CH_3_	H	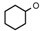	6.10
2	CH_2_CH(CH_3_)_2_	0	–	H	CH_3_	H	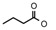	6.05
3^a^	CH_2_CH(CH_3_)_2_	0	–	H	CH_3_	H	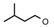	6.00
4	CH_2_CH(CH_3_)_2_	0	–	CH=CH_2_	CH_3_	H	OMe	6.00
5	CH_2_CH(CH_3_)_2_	0	–	H	CH_3_	H	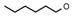	5.70
6	CH_2_CH(CH_3_)_2_	0	–	H	CH_3_	H	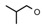	5.52
7	CH_2_CH(CH_3_)_2_	0	–	H	CH_3_	H	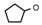	5.40
8^a^	CH_2_CH(CH_3_)_2_	0	–	H	CH_3_	H	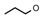	5.30
9	CH_2_CH(CH_3_)_2_	0	–	H	CH_3_	H	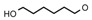	5.30
10	CH_2_CH(CH_3_)_2_	0	–	H	CH_3_	H	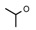	5.22
11	CH_2_CH(CH_3_)_2_	0	–	H	CH_3_	H	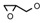	5.22
12^a^	CH_2_CH(CH_3_)_2_	0	–	H	CH_3_	H	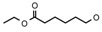	5.22
13	CH_2_CH(CH_3_)_2_	0	–	H	CH_3_	H	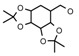	5.22
14	CH_2_CH(CH_3_)_2_	0	–	H	CH_3_	H	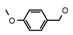	5.10
15	CH_2_CH(CH_3_)_2_	0	–	H	CH_3_	H	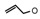	4.92
16	CH_2_CH(CH_3_)_2_	0	–	H	CH_3_	H	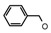	4.89
17	CH_2_CH(CH_3_)_2_	0	–	H	CH_3_	H	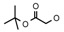	4.80
18	CH_2_CH(CH_3_)_2_	0	–	H	CH_3_	H	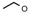	4.74
19	CH_2_CH(CH_3_)_2_	0	–	Br	CH_3_	Br	OMe	4.70
20	CH_2_CH(CH_3_)_2_	1	H	H	CH_3_	H	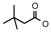	6.6
21	CH_2_CH(CH_3_)_2_	1	H	CH_3_	CH_3_	CH_3_	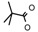	6.7
22^a^	CH_2_CH(CH_3_)_2_	1	H	H	CH_3_	H	(CH_3_)_2_CHSO_2_O	6.15
23^a^	CH_2_CH(CH_3_)_2_	1	H	Cl	CH_3_	Cl	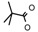	6.70
24	CH_2_C(CH_3_)_3_	1	H	H	CH_3_	H	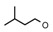	5.82
25	CH_2_CH(CH_3_)_2_	1	H	H	CH_3_	H	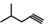	5.70
26	CH_2_CH(CH_3_)_2_	1	H	F	CH_3_	H	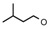	6.30
27^a^	CH_2_CH(CH_3_)_2_	1	H	H	CH_3_	H	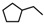	5.70
28	CH_2_CH(CH_3_)_2_	1	H	CN	CH_3_	H	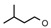	5.52
29	CH_2_CH(CH_3_)_2_	1	CH_3_	H	CH_3_	H	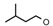	5.30
30	CH_2_CH(CH_3_)_2_	1	H	Br	CH_3_	Br	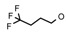	6.00
31	CH_2_CH(CH_3_)_2_	1	H	H	CH_3_	H	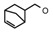	5.82
32^a^	CH_2_CH(CH_3_)_2_	1	H	H	CH_3_	H	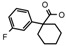	6.00
33	CH_2_CH(CH_3_)_2_	1	H	H	CH_3_	H	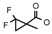	6.15
34	CH_2_CH(CH_3_)_2_	1	H	Cl	CH_3_	H	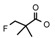	6.52
35	CH_2_CH(CH_3_)_2_	1	H	Cl	CH_3_	Br	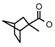	7.15
36	CH_2_CH(CH_3_)_2_	1	H	Cl	CH_3_	Br	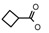	6.60
37	CH_2_CH(CH_3_)_2_	1	H	Br	CH_3_	Br	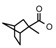	7.00
38	CH_2_CH(CH_3_)_2_	1	H	CH_3_	CH_3_	CH_3_	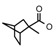	6.82
39^a^	CH_2_CH(CH_3_)_2_	1	H	Cl	CH_3_	Cl	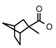	7.22
40^a^	CH_2_CH(CH_3_)_2_	1	H	Cl	CH_3_	Cl	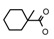	7.00
41	CH_2_CH(CH_3_)_2_	1	H	H	CH_3_	H	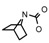	6.70
42	CH_2_CH(CH_3_)_2_	1	H	Cl	CH_3_	Cl	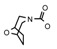	6.52
43	CH_2_CH(CH_3_)_2_	1	H	H	CF_2_H	H	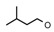	5.30
44	CH_2_CH(CH_3_)_2_	1	H	Br	CH_3_	Cl	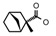	7.15
45	CH_2_CH(CH_3_)_2_	1	H	H	CH_3_	Cl	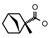	7.12
46	CH_2_CH(CH_3_)_2_	1	H	Cl	CH_3_	Br	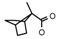	7.22
47	CH_2_CH(CH_3_)_2_	1	H	Cl	CH_3_	CH_3_	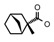	7.30
48	CH_2_CH(CH_3_)_2_	1	H	Cl	CH_3_	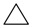	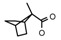	7.24
49^a^	CH_2_C(CH_3_)_3_	1	H	Cl	CH_3_	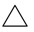	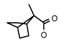	7.22
50	CH_2_C(CH_3_)_3_	1	H	Cl	CH_3_	Br	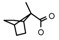	7.49
51	CH_2_CH(CH_3_)_2_	1	H	Cl	CH_3_	CH_2_OCH_3_	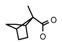	7.30
52	CH_2_CH(CH_3_)_2_	1	H	Cl	CH_3_	Br	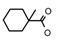	7.05
53	CH_2_C(CH_3_)_3_	1	H	Cl	CH_3_	CH_2_OCH_3_	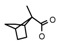	7.40
54	CH_2_CH(CH_3_)_2_	1	H	Cl	CH_3_	CH_3_	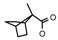	7.52
55	CH_2_C(CH_3_)_3_	1	H	Cl	CH_3_	CH_2_CH_3_	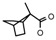	7.82
56	CH_2_CH(CH_3_)_2_	1	H	CH_3_	CH_3_	CH_3_	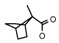	7.22
57	CH_2_CH(CH_3_)_2_	1	H	H	CH_3_	CH_2_CH_3_	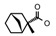	7.40
58	CH_2_CH(CH_3_)_2_	1	H	Cl	CH_3_	CH_2_CH_3_	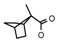	7.24
59	CH_2_CH(CH_3_)_2_	1	CH_3_	H	CH_3_	H	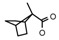	7.00
60	CH_2_CH(CH_3_)_2_	1	H	F	CH_3_	CH_3_	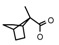	7.26
61	CH_2_CH(CH_3_)_2_	1	H	H	CH_3_	H	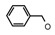	4.89
62	CH_2_CH(CH_3_)_2_	1	H	H	CH_3_	H	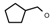	5.82
63	CH_2_CH(CH_3_)_2_	1	H	H	CH_3_	H	CF_3_SO_2_O	5.00
64	CH_2_CH(CH_3_)_2_	1	H	H	CH_3_	H	CH_3_(CH_2_)_4_SO_2_O	5.40
65	CH_2_CH(CH_3_)_2_	1	H	H	CH_3_	H	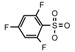	5.22
66	CH_2_CH(CH_3_)_2_	1	H	H	CH_3_	H	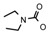	5.52
67	CH_2_CH(CH_3_)_2_	1	H	H	CH_3_	H	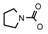	6.10
68	CH_2_CH(CH_3_)_2_	1	H	H	CH_3_	H	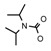	6.15
69	CH_2_CH(CH_3_)_2_	1	H	H	CH_3_	H	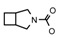	6.40
70^a^	CH_2_CH(CH_3_)_2_	1	H	H	CH_3_	H	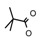	6.10
71	CH_2_CH(CH_3_)_2_	1	H	H	CH_3_	H	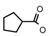	6.22
72	CH_2_CH(CH_3_)_2_	1	H	H	CH_3_	H	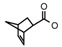	6.70
73^a^	CH_2_CH(CH_3_)_2_	1	H	H	CH_3_	H	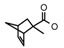	6.70
74^a^	CH_2_CH(CH_3_)_2_	1	H	H	CH_3_	H	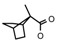	6.70

^a^ Table note: these compounds are used as a test set.

### Conformational analysis and alignment

Bioactive conformations and molecular alignment are two vital parameters to construct more reliable CoMFA and CoMSIA models. Unfortunately, to date, neither the crystal structure of a ligand-receptor complex nor the identification of a specific active site is available for these lactone-based analogues. As such, we chose the most potent compound **55** (IC­_50_ = 15 nM), as a template in this section.

The conformations of computationally energy-minimized molecules generally depend on the initial conformation. Additionally, a ligand may not bind to the receptor in the energy-minimized conformation, and instead, a certain degree of torsional freedom may be required to yield a lower energy ligand-receptor complex [15,16]. Here, simulated annealing was applied at a high temperature (*e.g.*, 1000 K) to overcome torsional energy barriers providing access to alternate low-energy conformations.

First, an initial geometry optimization was performed using Powell method (the Tripos force field, Gasteriger-Hückel charges, 1000 iterations, and an energy convergence cutoff of 0.001 kcal·mol^-1^·Å^-1^). Next, simulated annealing was conducted by heating at an initial temperature of 1000 K for 1000 fs, and then cooling to 250 K within 1500 fs of annealing time. The exponential annealing function was utilized, and 10 cycles were conducted. Next, the conformations at 250–300 K were calculated by hierarchical clustering in the Advanced CoMFA module. The lowest energy conformer in each larger cluster was selected for further minimization as described above. Finally, all minimized conformers were superimposed by the SYBYL Matchfit function, and the most similar conformer to the others was chosen as a template.

**Figure 8:**
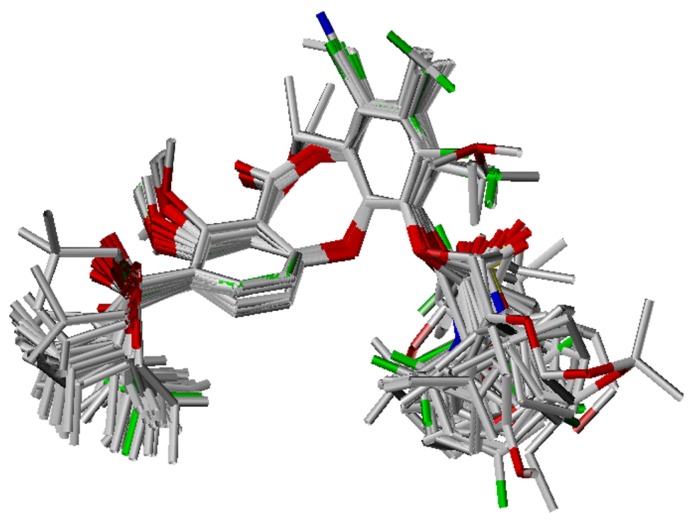
Alignment of the lactone-based analogues from the training set.

The remaining molecules were generated based on the template conformation, and then optimized as described. These were then superimposed onto the template on the dibenzodioxocinone backbone, and a proper conformer was determined according to alignment (Figure 8).

### CoMFA study

CoMFA calculates steric fields using a Lennard-Jones potential and electrostatic fields using a Coulombic potential [17]. To improve the signal-to-noise ratio, a variable column filtering energy cutoff was set at 2.09, 4.18, 6.27, 8.63, 10.45, and 12.54 kcal/mol. Several probes (C.3, O.3, H, N.3, and N.pl^3^) and grid spacings (1.0, 1.5, 2.0, 2.5, and 3.0 Å) were taken into account as well. On the basis of the above optimal parameters, AOS and APS were executed by rotating and translating the molecular aggregate within the grid [18], and an orientation that yielded the highest q^2^ value was selected. Next, the model was optimized by altering steric and electrostatic field cutoffs (20.9–146.3 kcal/mol). Finally, region focusing was utilized to improve the predictability of the model further. 

### CoMSIA study

CoMSIA employs a Gaussian function that is used to measure the distance dependence between a probe atom and molecular atoms [19,20]. To make a valid comparison with the CoMFA model, we used the optimal model obtained by AOS/APS to systematically investigate the effect of different attenuation factors (0.2, 0.3, and 0.4) at different column filtering energy cutoffs between 2.09 and 12.54 kcal/mol. Finally, as in the CoMFA study, various grid spacings from 1.0 Å to 3.0 Å and region focusing were investigated.

### EVA study

The EVA descriptor is derived from calculated fundamental IR- and Raman-range molecular vibrational frequencies, typically obtained through the application of a normal coordinate analysis (NCA) to an appropriately energy minimized structure [21,22,23]. For a compound with N atoms there are 3N-6 (or 3N-5 for a linear structure) normal modes of vibration. The frequency set for a given structure is projected onto a bounded frequency scale (BFS) covering a range from 0 to 4000 cm^-1^. Next a Gaussian kernel of fixed standard deviation (σ) is placed over each and every frequency value. The BFS is then sampled at fixed increments of δ cm^-1^ and the value of the resulting EVA descriptor at sample point x, EVA_x_, is the sum of the amplitudes of the overlaid kernels at that point:

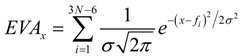

where f_i_ is the ith normal mode frequency of the compound concerned. This procedure is repeated for each dataset compound and results in a descriptor set consisting of 4000/δ variables. EVA descriptors are independent of the orientation of the molecules in space; just sensitive to 3D structure. So the studies started with conformations previously converted by AOS/APS, and the default setting (σ = 10 cm^-1^; δ = 5 cm^-1^; AM1; 200-4000 cm^-1^) was used for defining EVA profiles.

### PLS analysis and validation

A partial least-squares (PLS) approach was applied to derive the 3D-QSAR, employing CoMFA, CoMSIA and EVA descriptors as independent variables, and pIC_50_ values as dependent variables. To measure the internal predictive ability of the derived model, cross-validations were conducted through the leave-one-out procedure. The optimal number of components was determined in such a manner that each additional component increased the q^2^ value (cross-validated r^2^ value) by at least 5%. The final PLS analysis was conducted without cross-validation with an optimum number of components reported from the cross-validation results. Furthermore, bootstrapping analysis [24,25,26] was performed for 200 runs in order to estimate the confidence limits for the parameters. Additionally, progressive scrambling [27] was conducted for the evaluation of the sensitivity of a QSAR model to chance correlations. For most models, if the number of scramblings is greater than 30, the dependence on random number seed is not large enough to affect the outcome. Therefore, the number of scramblings was set to 40 and the seed value to 123456. Finally, to test the external predictivity of the final model, predictive r^2^ values were calculated on the test set using the following equation:

r^2^_pred_ = 1 – (PRESS/SSD)

where PRESS is the sum of the squared differences between the observed activities and predicted activities, and SSD is the sum of the squared differences between the measured activities of the test set and average measured activity of the training set.

## Supplementary data

The experimental and predicted values for the training set in the CoMFA, CoMSIA, and EVA models are available in Table S1.

## References

[1] Wolfe M.L., Rader D.J. (2004). Cholesteryl Ester Transfer Protein and Coronary Artery Disease: An Observation with Therapeutic Implications. Circulation.

[2] Barter P.J., Brewer H.B., Chapman M.J., Hennekens C.H., Rader D.J., Tall A.R. (2003). Cholesteryl Ester Transfer Protein: A Novel Target for Raising HDL and Inhibiting Atherosclerosis. Arterioscler. Thromb. Vasc. Biol..

[3] Ruggeri R.B. (2005). Cholesteryl Ester Transfer Protein: Pharmacological Inhibition for the Modulation of Plasma Cholesterol Levels and Promising Target for the Prevention of Atherosclerosis. Cur. Top. Med. Chem..

[4] Cuchel M., Rader D.J. (2007). Is the Cholesteryl Ester Transfer Protein Proatherogenic or Antiatherogenic in Humans?. J. Am. Coll. Cardiol..

[5] Parini P., Rudel L.L. (2003). Is There a Need for Cholesteryl Ester Transfer Protein Inhibition?. Arterioscler. Thromb. Vasc. Biol..

[6] Eary C.T., Jones Z. S., Groneberg R.D., Burgess L.E., Mareska D.A., Drew M.D., Blake J.F., Laird E.R., Balachari D., O'Sullivan M., Allen A., Marsh V. (2007). Tetrazole and ester substituted tetrahydoquinoxalines as potent cholesteryl ester transfer protein inhibitors. Bioorg. Med. Chem. Lett..

[7] Brückner D., Hafner F.-T., Li V., Schmeck C., Telser J., Vakalopoulos A., Wirtz G. (2005). Dibenzodioxocinones-A new class of CETP inhibitors. Bioorg. Med. Chem. Lett..

[8] Brousseau M.E., Schaefer E.J., Wolfe M.L., Bloedon L.T., Digenio A.G., Clark R.W., Mancuso J.P., Rader D.J. (2004). Effects of an Inhibitor of Cholesteryl Ester Transfer Protein on HDL Cholesterol. N. Engl. J. Med..

[9] Reinhard E.J., Wang J.L., Durley R.C., Fobian Y.M., Grapperhaus M.L., Hickory B.S., Massa M.A., Norton M.B., Promo M.A., Tollefson M.B., Vernier W.F., Connolly D.T., Witherbee B.J., Melton M.A., Regina K.J., Smith M.E., Sikorski J.A. (2003). Discovery of a Simple Picomolar Inhibitor of Cholesteryl Ester Transfer Protein. J. Med. Chem..

[10] Okamoto H., Fumlhiko Y., Korekiyo W., Takashi M. (2000). A cholesteryl ester transfer protein inhibitor attenuates atherosclerosis in rabbits. Nature.

[11] Tomoda H., Matsushima C., Tabata N., Namatame I., Tanaka H., Bamberger M.J., Arai H., Fukazawa M., Inoue K., Omura S. (1999). Structure-specific inhibition of cholesteryl ester transfer protein by azaphilones. J. Antibiot..

[12] Krishna R., Anderson M.S., Bergman A.J., Jin B., Fallon M., Cote J., Rosko K., Chavez-Eng C., Lutz R., Bloomfield D.M., Gutierrez M., Doherty J., Bieberdorf F., Chodakewitz J., Gottesdiener K.M., Wagner J.A. (2007). Effect of the cholesteryl ester transfer protein inhibitor, anacetrapib, on lipoproteins in patients with dyslipidaemia and on 24-h ambulatory blood pressure in healthy individuals: two double-blind, randomised placebo-controlled phase I studies. Lancet.

[13] Qiu X., Mistry A., Ammirati M.J., Chrunyk B.A., Clark R.W., Cong Y., Culp J.S., Danley D.E., Freeman T.B., Geoghegan K.F., Griffor M.C., Hawrylik S.J., Hayward C.M., Hensley P., Hoth L.R., Karam G.A., Lira M.E., Lloyd D.B., McGrath K.M., Stutzman-Engwall K.J., Subashi A.K., Subashi T.A., Thompson J.F., Wang I.-K., Zhao H., Seddon A.P. (2007). Crystal structure of cholesteryl ester transfer protein reveals a long tunnel and four bound lipid molecules. Na. Struct. Mol. Biol..

[14] Bayer HealthCare AG Patent Appl. WO.

[15] Jain A.N., Koile K., Chapman D. (1994). Compass: predicting biological activities from molecular surface properties, performance comparisons on a steroid benchmark. J. Med. Chem..

[16] Bush A., Martin-Pastor M. (1999). Structure and conformation of complex carbohydrate of glycoproteins, glycolipids, and bacterial polysaccharides C. Annu. Rev. Biophys. Biomol. Struct..

[17] Cramer R.D., Patterson D.E., Bunce J.D. (1988). Comparative molecular field analysis (CoMFA). 1. Effect of shape on binding of steroids to carrier proteins. J. Am. Chem. Soc..

[18] Wang R.X., Gao Y., Liu L., Lai L.H. (1998). All-orientation search and all-placement search in comparative molecular field analysis. J. Mol. Model..

[19] Klebe G., Abraham U., Mietzner T. (1994). Molecular Similarity Indices in a Comparative Analysis (CoMSIA) of Drug Molecules to Correlate and Predict their Biological Activity. J. Med. Chem..

[20] Klebe G., Abraham U. (1999). Comparative Molecular Similarity Index Analysis (CoMSIA) to Study Hydrogen Bonding Properties and to Score Combinatorial Libraries. J. Comput.-Aided Mol. Des..

[21] Ferguson A.M., Heritage T., Jonathon P., Pack S.E., Philips L. (1997). EVA: A New Theoretically Based Molecular Descriptor for Use in QSAR/QSPR Analysis. J. Comput.-Aided. Mol. Des..

[22] Makhija M.T., Kulkarni V.M. (2001). Eigen Value Analysis of HIV-1 Integrase Inhibitors. J. Chem. Inf. Comput. Sci..

[23] Turner D.B., Willett P., Ferguson A.M., Heritage T.W. (1999). Evaluation of a Novel Molecular Vibration-Based Descriptor (EVA) for QSAR Studies. 2. Model Validation Using a Benchmark Steroid Dataset. J. Comput.-Aided. Mol. Des..

[24] Agrafiotis D.K., Cedeno W., Lobanov V.S. (2002). On the use of neural network ensembles in QSAR and QSPR. J. Chem. Inf. Comput. Sci..

[25] Cramer R.D., Bunce J.D., Patterson D.E., Frank I.E. (1988). Crossvalidation, bootstrapping, and partial least squares compared with multiple regression in conventional QSAR studies. Quant. Struct.-Act. Relat..

[26] Wehrens R., van der Linden W.E. (1997). Bootstrapping principal component regression models. J. Chemom..

[27] Clark R.D., Sprous D.G., Leonard J.M., Höltje H. D., Sippl W. (2001). Validating models based on large data sets. Rational Approaches to Drug Design.

